# Non-dipping and arterial hypertension depend on clinical factors rather than on genetic variability of *ACE* and *RGS2* genes in patients with type 1 diabetes

**DOI:** 10.1007/s00592-014-0568-0

**Published:** 2014-02-23

**Authors:** G. Deja, M. Borowiec, W. Fendler, I. Pietrzak, A. Szadkowska, L. Machnica, J. Polanska, W. Mlynarski, P. Jarosz-Chobot

**Affiliations:** 1 Department of Pediatrics, Pediatric Endocrinology and Diabetology, Medical University of Silesia, Medykow 16 Str., 40-752 Katowice, Poland; 2 Department of Pediatrics, Oncology, Hematology and Diabetology, Medical University of Lodz, Lodz, Poland; 3 Department of Pediatrics, Pediatric Endocrinology and Diabetology, Upper Silesia Child Health Center, Katowice, Poland; 4 Faculty of Automatic Control, Electronics and Computer Science, Silesian University of Technology, Gliwice, Poland

**Keywords:** Diabetes mellitus type 1, Hypertension, Non-dipping, ABPM, *ACE* genotype, *RGS2* genotype

## Abstract

The aim of our study was to characterize the association of clinical and genetic risk factors such as: *ACE* genotype (rs17997552, rs1800764, rs4459609) and *RGS2* (rs2746071) with the development of hypertension (HT) and non-dipping phenomenon in patients with type 1 diabetes mellitus (T1DM). A total of 238 adolescents and young adults with T1DM—103 females and 135 males, aged 8–30 years (mean 17.35 ± 5.2) with diabetes duration 1–26 years (mean 7.72 ± 6.2), with mean HbA1c (IFCC) 58 ± 15 mmol/mmol—were subjected to 24-h ambulatory blood pressure measurements (ABPM). The results of the ABPM were analyzed in association with the polymorphisms of *ACE* and *RGS2* genes and clinical data of patients. HT was recognized in 65 (27 %) and non-dipping in 111 (46.63 %) patients. In the multivariate analysis of factors predisposing to HT, the variables that remained significant were the following: male sex (OR 1.62; 95 % CI 1.171–2.250), non-dipping (OR 1.40; 95 % CI 1.03–1.90) and total cholesterol level (OR 1.01; 95 % CI 1.005–1.021). The only factor influencing non-dipping was the duration of diabetes—OR 1.09 (95 % CI 1.04–1.14). The patients displaying non-dipping have a twice increased risk of development of HT (OR 2.17; 95 % CI 1.21–3.89). There was no association between disturbances of blood pressure (BP) and genotypes of *ACE*: rs17997552, rs1800764, rs4459609 and *RGS2*: rs2746071. Clinical rather than genetic risk factors seem to be connected with BP disturbances in young patients with T1DM. Although we have identified representative groups of HT versus non-HT and dipping versus non-dipping subjects, the effect of genetic predisposition to the development of higher BP is too weak to be statistically significant.

**Electronic supplementary material:**

The online version of this article (doi:10.1007/s00592-014-0568-0) contains supplementary material, which is available to authorized users.

## Introduction

Despite improved methods of treatment and monitoring, type 1 diabetes mellitus (T1DM) still remains a disease with high risk of chronic micro- and macrovascular complications. One of the most important risk factors contributing to these complications is hypertension (HT). The most important traditional risk factors connected with the development of HT are as follows: overweight/obesity, atherogenic lipid profile, and in patients with diabetes, additionally the following: poor glycemic control and long duration of diabetes [1, 2].

Genetic approaches may provide a powerful tool for explaining the etiology and pathogenesis of HT. Many studies in the previous years have confirmed that the genetic variants of major components of the renin-angiotensin system (RAS) are associated with higher blood pressure (BP), myocardial infarction and lacunar stroke in non-diabetic patients [3, 4]. In diabetic patients, especially children with diabetes, the evidence is not clear. Some studies have shown that different polymorphisms in angiotensin 1-converting enzyme (ACE) gene are associated with development of both persistent microalbuminuria and diabetic nephropathy [5, 6]; however, other recently published reports have not replicated these findings [7, 8]. Furthermore, a few studies suggested an association between *ACE* genotype and tendency to higher BP in normoalbuminuric, normotensive patients during ABPM measurements in T1DM children [9, 10], but to date, there are no studies exploring this in adolescents and young adults with HT and diabetes.

Additionally, BP homeostasis is very precisely regulated through hormones and neurotransmitters activating G-protein-coupled receptors. These mechanisms control, among other things, the diameter of resistance arterioles as well as the electrolyte and fluid excretion rates in the kidney [11, 12]. Recently, a major regulator of G-protein signaling (RGS), RGS2 protein, was identified and strongly associated with HT. It was demonstrated that mice lacking RGS2 developed strong HT and persistently increased vascular tone [11]. Subsequently, two studies described the hypertensive phenotype associated with *RGS2* in humans, but there is no evidence of its significance in patients with T1DM [13, 14].

The aim of our study was to characterize the association of chosen clinical risk factors and genotypes with the development of HT and non-dipping phenomenon in children and young adults with T1DM. We hypothesized that the genetic variations in the *ACE* genotype—insertion/deletion (rs17997552), rs1800764, rs4459609—and *RGS2* rs2746071 genotype could be connected with the regulation of the BP and lead to its disturbances.

## Methods

### Study population

A total of 238 adolescents and young adults with T1DM—103 females and 135 males, aged 8–30 years (mean 17.35 ± 5.2) with diabetes duration 1–26 years (mean 7.72 ± 6.2)—were included to the study. The study group was very diverse in terms of age and diabetes duration to assess the effect of genetic predisposition on developing BP disturbances. The study protocol was approved by the Local Ethics Committee. Informed consent was obtained from the parents of children and/or from children >16 years of age and adult patients themselves. The patients were recruited from nearly 1,800 diabetic patients regularly attending to the diabetic Outpatients Clinic in Katowice or Lodz. The Department of Pediatrics, Pediatric Endocrinology and Diabetology Medical University of Silesia in Katowice and Department of Pediatrics, Oncology, Hematology and Diabetology Medical University of Lodz in Lodz are the regional referral centers for children with T1DM. The centers are comparable with respect to both the amount of patients (about 900 in each) and the quality of diabetes care with the average of Hba1c about of 58 mmol/mmol for all patients [15]. The inclusion criteria for the study were as follows: diabetes recognition confirmed by positive antibody tests and diabetes duration above 1 year. The exclusion criteria were as follows: diabetic patients with other types of diabetes and other chronic diseases.

Subjects were randomly selected for the study during the routine annual check of diabetes care including blood tests and ambulatory blood pressure measurements (ABPM). All the patients were treated intensively by multiple injections of short-acting regular insulin/analog and long-acting NPH/long-acting analog insulin or by using insulin pumps. The patients and their parents have been repeatedly educated in diabetes care and have reached good/suboptimal long-term glycemic control, as measured by mean HbA1c since diabetes onset until the end of the study. HbA1c was analyzed as a continuous variable and as a dichotomous one, divided on a >75 mmol/mol cutoff, in order to specifically evaluate the impact of poor metabolic control.

### Study protocol

After the formal clerking, basic blood tests were performed. The information about the mean HbA1c [high-performance liquid chromatography (HPLC) method], lipid profile (enzymatic method) and daily insulin dose from the last visit was used for statistical analysis. HbA_1c_ assays were performed by ion-exchange HPLC using the Bio-Rad VARIANT™ Hemoglobin A1c Program (Bio-Rad Laboratories, Inc. Hercules, California, USA). The VARIANT™ Hemoglobin A1c Program has been certified by the National Glycohemoglobin Standardization Programme (NGSP) as meeting the DCCT standard. Reference values for healthy people estimated by the local laboratory were from 4.3 to 5.7 %. The within-run coefficient of variation (CV) determined by the manufacturer was 1.05 % for normal patients and 0.94 % for diabetic patients; the between-run CV was 1.61 and 1.16 % for normal and for diabetic patients, respectively, as reported previously [16]. In all patients, the estimation of albumin excretion rate (AER) was obtained from overnight urine sample. Microalbuminuria was defined as the presence of two consecutive specimens with AER of >20 and <200 μg/min, as recommended by International Society of Pediatric and Adolescents Diabetes (ISPAD).

The 24-h ambulatory blood pressure measurements (ABPM—Spacelabs Health, Model 90217, Hertford, UK) were carried out in each patient based on the oscillometric method with the appropriate cuff size. From 0600 to 2200 hours, BP was measured every 20 min and the night measurements from 2200 to 600 hours were performed every 30 min. Only records with a minimum 80 % of valid measurements were taken into account. The norm values were estimated individually for each adolescent based on the sex, age and height percentile norm [17]. The means of 24-h BP, day period, night period and diurnal variations of mean BP were calculated. HT was diagnosed when the mean 24-h systolic pressure (SP) and diastolic pressure (DP) was >95th percentile for sex, age and height of the pediatric subject and >140/90 in adults according the National High Blood Pressure Education Program Working Group on High Blood Pressure in Children and Adolescents [17]. Furthermore, diurnal variations of BP were estimated and patients whose mean BP during the day decreased by <10 % in the night were defined as non-dipping. We verified the abnormal results: by the following up another ABPM and by collecting the measurements using the traditional oscillometric method performed in the hospital and/or at home by patients/parents. Pubertal status was evaluated using the Tanner stage, with a Tanner stage 1 considered prepubertal, stages 2–4 pubertal and stage 5 as postpubertal.

### Genetic tests

#### Isolation of DNA

A volume of 3 ml of peripheral blood was collected in sterile tubes with anticoagulant 10 % EDTA (pH 8.0). DNA was isolated using a commercially available kit column AxyPrep Blood Genomic DNA Miniprep Kit (Axygen, USA).

A volume of 250 μl of blood was added up to 500 μl of Buffer AP1. The mixture was strongly shaken and then added to 100 μl of AP2 buffer and centrifuged for 10 min. 12,000×*g* at room temperature. After centrifugation, the supernatant was transferred to a column miniprep, centrifuged and then washed with WIA Buffer (700 μl) and twice with W2 Buffer (800 and 500 μl). After drying column (12,000×*g*, 1 min), to elute the genomic DNA, 200 μl of TE buffer was added and next centrifuged 12,000×*g* for 1 min. The purified DNA was stored at −20 °C.

#### Analysis of the polymorphisms

Polymorphisms of genes implicated in BP regulation were analyzed (Table 1). These polymorphisms were selected for the study based on evidence from the literature. The best known gene variant association in the *ACE* genes is I/D variant, with allele DD connected with potentially higher BP [4, 6, 9, 10]. Moreover, we chose to study another two single nucleotide polymorphisms (SNP) of *ACE*, rs1800764 and rs4459609, because they were shown to characterize the haplotypic structure of *ACE* in European populations [18, 19]. Additionally, we chose the hypertensive phenotype of *RGS2* gene described by Sugimoto et al [12] in Japanese population to replicate their findings in the Polish patients with DMT1.

**Table 1:** The selected polymorphisms

Gene	Polymorphisms	rs
*ACE*_1 C___1838552_10	transitions C → T (C/T) at position −3903 in the promoter sequence	rs1800764
*ACE*_2 C___1838551_10	transitions A → C (A/C) at position −5,486 in the promoter sequence	rs4459609
*ACE*_ID	Ins/del 50 pair at position −3,678 in the intron sequence	rs1799752
*RGS2* C___2498711_20	transitions A → G (A/G) at position −638 in the promoter sequence	rs2746071

#### Genotyping of polymorphic sites selected genes

For the analysis of three SNPs (*ACE*, *RGS2*), optimized reagent system was used to study point mutations of genes—TaqMan ^®^ SNP Genotyping Assays (Applied Biosystems, USA). The reactions were carried out using an Applied Biosystems thermocycler 7900HT Fast Real-Time PCR, the 96-well plates in a volume of 5 μl. The components of the reaction mixture of real-time PCR were, in addition to fluorescent dye-labeled probe, appropriately selected primers, DNA polymerase enzyme and the DNA matrix. Moreover, the reaction buffer containing the enzyme necessary for the working of metal ions and diphosphates of deoxyribonucleotides (dNTPs) was added to the mixture. *ACE* polymorphisms (insertion/deletion of 50 nucleotide pairs) were identified in horizontal electrophoresis in 2 % agarose gel using QantityOne software (BioRad, USA).

### Statistical analysis

Categorical data were compared using the chi-squared test or, if the number of patients in any of the compared groups was lower than five, using the two-tailed Fisher’s exact test. Comparisons between two groups were performed using Student’s *t* test. Analysis of variance was used for comparisons of more than two groups. Multivariate analyses were performed using logistic regression. Variable selection was performed using backward stepwise modeling with a *p* value for variable exclusion of 0.15. Genetic factors were forced to be retained in the model regardless of significance in univariate analysis. A *p* value of <0.05 was considered as statistically significant.

## Results

Based on ABPM records, HT was recognized in 65 (27 %) patients, including persistent HT, recognized earlier treated with hypertensive drugs (ACE inhibitors) in 9 patients and HT as a new diagnosis in the remaining patients. The characteristics of the subjects with and without HT are shown in the Table 2. Subjects with HT were more frequently male, had a longer duration of diabetes and had worse lipid profiles with significantly higher levels of total and LDL cholesterol and marginally higher triglyceride levels. Subjects classified as having HT tended to be older (however, this trend did not reach significance). We found no differences in HbA1c, BMI, daily insulin dose and the presence of microalbuminuria between the hyper- and normotensive groups, which could have been a result of a relatively good metabolic control in all of the studied subjects.

**Table 2:** Characteristics of the subjects: the whole group and subgroups: hypertension/normal BP and non-dippers/dippers concerning clinical data and the results of the laboratory tests

	Total *N* = 238	Hypertension versus normal BP			Non-dippers versus dippers		
		HT (+) *(N* = 65)	HT (−) *(N* = 173)	P	Non-dippers *(N* = 111)	Dippers *(N* = 127)	*P*
F/M	103/135	20/45	83/90	0.01698	46/65	57/70	0.59
Age (years)	17.35 ± 5.20	18.67 ± 6.33	16.86 ± 4.62	0.094787	18.64 ± 5.49	16.22 ± 4.67	0.002172
Diabetes duration (years)	7.72 ± 6.23	9.72 ± 6.68	6.97 ± 5.91	0.002010	9.42 ± 6.65	6.23 ± 5.46	0.000256
BMI (normal/overweigh/obese)	175/42/21	44/12/9	131/30/12	0.22	78/23/10	97/19/11	0.49
Total cholesterol (mg/dl)	177.63 ± 39.36	190.63 ± 43.43	172 ± 36.67	0.005492	182.21 ± 42.76	173.63 ± 35.83	0.231844
LDL cholesterol (mg/dl)	96.98 ± 32.63	106.75 ± 34.17	93.31 ± 31.35	0.004647	100.20 ± 36.54	94.16 ± 28.63	0.442408
HDL cholesterol (mg/dl)	63.15 ± 16.50	63.24 ± 14.09	63.11 ± 17.35	0.732890	63.72 ± 17.29	62.65 ± 15.82	0.751899
Triglyceride (mg/dl)	88.30 ± 50.07	94.64 ± 55.04	85.92 ± 48.02	0.073305	91.81 ± 52.99	85.24 ± 47.36	0.226357
Microalbuminuria (positive/negative in %)^a^	14/186	9/91	5/95	0.6965	2/98	8/92	0.0590
HbA1c IFCC mean (mmol/mmol)	58 ± 15	59 ± 16	58 ± 14	0.925898	58 ± 14	58 ± 15	0.651159
Daily insulin dose (u/kg/day)	0.82 ± 0.23	0.85 ± 0.15	0.81 ± 0.25	0.1633	0.85 ± 0.23	0.80 ± 0.23	0.1030
Pubertal status (prepubertal/pubertal/postpubertal)	20/85/133	6/22/37	14/63/96	0.9139	5/39/67	15/46/66	0.1080
Dipping (yes/no)	127/111	26/39	101/72	0.0113	NA	NA	NA

*NA* not applicable

^a^unknown in 38 cases

Non-dipping was frequent in the studied group and was reported in 111 (46.63 %) patients. Similarly, the analysis of diurnal variables of BP showed that subjects with non-dipping phenomenon were significantly older and had a longer duration of T1DM. There were no associations between dipping and other analyzed clinical parameters (Table 2). Patients who did not display dipping showed a twofold increase in risk of developing HT (OR = 2.17; 95 % CI 1.21–3.89).

There were no differences among *ACE* genotypes at all three analyzed loci (rs17997552, rs1800764, rs4459609) and *RGS2* genotype (rs2746071) in terms of HT and diurnal variations of BP. The distribution of the analyzed loci in relation to HT and non-dipping are presented in the Table 3.

**Table 3:** *ACE* and *RGS2* genotypes of participants in relation to HT and to non-dipping (*p* = NS)

		Hypertension versus normal BP		*P* value	Non-dippers versus dippers		*P* value
		HT (+) *(N* = 65)	HT (−) *(N* = 173)		Non-dippers *(N* = 111)	Dippers *(N* = 127)	
*ACE* rs1800764	TT	22 (34 %)	55 (30 %)	0.8958	37 (33 %)	38 (30 %)	0.8479
	CT	29 (45 %)	84 (49 %)		51 (46 %)	62 (49 %)	
	CC	14 (21 %)	36 (21 %)		23 (21 %)	27 (21 %)	
*ACE* rs4459609	AA	25 (39 %)	60 (35 %)	0.8479	39 (35 %)	46 (36 %)	0.7153
	AC	30 (46 %)	83 (48 %)		51 (46 %)	62 (49 %)	
	CC	10 (15 %)	30 (17 %)		21 (19 %)	19 (15 %)	
*ACE* I/DD rs1799752	II	16 (25 %)	40 (23 %)	0.5194	25 (23 %)	31 (24 %)	0.8825
	ID	19 (29 %)	64 (37 %)		38 (34 %)	45 (36 %)	
	DD	30 (46 %)	69 (40 %)		48 (43 %)	51 (40 %)	
*RGS2* rs2746071	AA	34 (52 %)	102 (59 %)	0.4404	62 (56 %)	74 (58 %)	0.5516
	AG	23 (36 %)	58 (34 %)		41 (37 %)	40 (32 %)	
	GG	8 (12 %)	13 (8 %)		8 (7 %)	13 (10 %)	

We found no evidence of association between any of the analyzed genotypes and percentages of abnormally high SBP or DBP values for both day and night (Table 4).

**Table 4:** Association between genotypes and blood pressure load

	*ACE* (rs1800764)			*P* value
	TT	CT	CC	
Daytime				
SBP % up	5 (0–32)	8 (0–24)	5 (0–25)	0.9793
DBP % up	4 (0–13)	3 (0–12)	2 (0–9)	0.4926
Nighttime				
SBP % up	8 (0–47)	8 (0–40)	12 (0–33)	0.7812
DBP % up	0 (0–9)	0 (0–13)	0 (0–13)	0.9555

	*ACE* (rs4459609)			*P* value
	AA	AC	CC	
Daytime				
SBP % up	6 (0–32)	6 (0–24)	6 (0–24)	0.9184
DBP % up	3 (0–15)	3 (0–10)	3 (0–10)	0.7331
Nighttime				
SBP % up	8 (0–40)	8 (0–41)	10 (0–35)	0.9982
DBP % up	0 (0–14)	0 (0–10)	5 (0–13)	0.2007

	*ACE* (rs1799752)			*P* value
	DD	ID	II	
Daytime				
SBP % up	7 (0–27)	3 (0–24)	7 (0–23)	0.3412
DBP % up	5 (0–13)	2 (0–11)	3 (0–11)	0.2382
Nighttime				
SBP % up	8 (0–47)	8 (0–31)	8 (0–40)	0.8030
DBP % up	0 (0–14)	0 (0–10)	0 (0–10)	0.7227

	*RGS2* (rs2746071)			*P* value
	AA	AG	GG	
Daytime				
SBP % up	7 (0–27)	5 (0–23)	6 (0–36)	0.7792
DBP % up	3 (0–12)	3 (0–10)	7 (0–24)	0.7840
Nighttime				
SBP % up	8 (0–33)	8.5 (0–45)	9 (0–47)	0.9773
DBP % up	0 (0–10)	0 (0–12)	7 (0–20)	0.3425

Values within the table represent the percentages of daytime and nighttime systolic and diastolic blood pressure above normal range for age, sex and height percentile

In multivariate analysis of factors predisposing to HT, the variables that remained significant were the following: male sex, non-dipping and total cholesterol level (Table 5.). Even after correction for these variables, none of the genetic factors showed any trend for significance toward association with HT. Multivariate analysis of factors associated with the presence of non-dipping phenomenon showed that the only factor which was retained in the model was the duration of diabetes—OR 1.09 (95 % CI 1.04–1.14; *p* = 0.0001). None of the analyzed genetic polymorphisms showed any significant relation to non-dipping. The impact of HbA1c was not statistically significant in models testing the variable as a continuous or dichotomous factor (all *p* values >0.5).

**Table 5:** Multivariate analysis of factors predisposing to HT

	OR	95 % CI	*p*
Male gender	1.623	1.171–2.250	0.003644
Non-dipper	1.402	1.034–1.902	0.029581
Total cholesterol	1.013	1.005–1.021	0.001099

## Discussion

The rapidly increasing morbidity of diabetes among children in the last years and estimated continuation of this trend in the future makes the studies concerning risk factors for chronic complications extremely valuable. Indication of the group with high risk of HT, especially in the pediatric population, will give the chance for early prevention and treatment. It is commonly known that diabetes and increased BP potentiate each other as risk factors for severe cardiovascular complications.

Our study confirmed that the most important clinical risk factors for disturbances with BP in children and young adults with DMT1 remain to be male sex, duration of diabetes and atherogenic lipid profile. This was confirmed both in the univariate analysis and also in multivariate analysis with logistic regressions. Patients that did not display a BP dip in the night were shown to have twice increased risk of development of HT. These agents are well-known parameters connected with HT, as well as identified risk factors for endothelium damage and macroangiopathy [1, 20, 21]. In the recently published study, Lee et al. [22] have shown that especially nocturnal HT is associated with increased carotid intima-media thickness (cIMT). In Lee’s study, the cIMT was significantly higher in the hypertensive group than in the non-hypertensive group, and HDL cholesterol concentration was significantly lower in the hypertensive group than in the non-hypertensive group. Thus, ABPM may be a useful method for detecting the macrovascular complications of DMT1.

In the studies concerning the development of HT in diabetic patients, clinical factors, such as poor metabolic control, high daily insulin dose and overweight/obesity, are usually, but not always, implicated as causative [23–25]. In the present study, we have not found such an association, probably because our patients were generally well/suboptimally controlled. They have been intensively treated with multiple injections or insulin pumps from the onset of diabetes and extensively educated about the importance of low insulin dosing, as is the standard of care in our centers. In our study, the overweight/obese was observed more frequently in the HT versus non-HT group (32 vs. 25 %), but the differences were not significant. In our previous study concerning diabetic normotensive patients, we have noted positive correlations between mean BP and both BMI and body composition—fat mass content [25]. Although BMI is commonly accepted as an easily available indicator of obesity, we should remember that it depends on not only fat but also on muscle mass. In the present study, we have the predominance of hypertensive males, so we can suspect that some of the older adolescents and young men have higher BMI because of well developed muscles. In this situation, our result should be treated with a caution.

The group of patients selected for this study was very diverse in terms of age, gender and disease duration, with confirmed clinical characteristics of HT present among some of the subjects. We believe therefore that our subject group was appropriately representative for genetic studies. In our study, we have hypothesized that the genetic predisposition for development of HT could be recognized from the beginning of diabetes. We have assessed the distribution of genes connected with increased risk of higher BP, which we hypothesized would be observed more frequently among the subjects with recognized/treated HT. The *ACE* genes have been assessed in relation to HT and diabetic nephropathy for many years, but the results of these studies are conflicting. The best known gene variant association is the I/D polymorphism, in which DD allele determines higher angiotensin level and therefore potentially higher BP [18, 26]. This observation has been confirmed in many studies conducted in the general population and among subjects with T2DM [6, 27, 28]. Studies performed in the pediatric population have shown that allele DD in *ACE* polymorphism is associated with higher BP in normotensive, normoalbuminuric diabetic children in ABPM measurements [9, 29]. Similarly in our previous study, when we have taken into account only clinically normotensive and normoalbuminuric children, we have proven that subjects in the DD genotype group had significantly higher nocturnal BP in comparison with ID/II genotype group, and this was also connected with lower dipping [10]. However, in the present study, where we have analyzed the BP in the group with HT and without HT, we have not found a more frequent occurrence of the DD genotype among patients with HT and non-dipping. Our study is in agreement with the results of other researches, who did not replicate the associations between the genotype of *ACE* genes and HT in children with T1DM [7, 8].

In addition to the I/D variant which was widely investigated, we chose to study another two SNP of *ACE*, rs1800764 and rs4459609, because they were shown to characterize the haplotypic structure of *ACE* in European populations [25, 29]. However, to our knowledge, there had not been any clinical studies in humans concerning these SNPs in relation to BP. Our study has not confirmed any association of the considered SNPs with HT or non-dipping in the studied group of adolescents and young adults with T1DM. An important point of our study was to analyze the *RGS2* gene polymorphisms in association with BP regulation, as there is very little data on the topic. Genetic changes in *RGS2* have been proven to be associated with a hypertensive phenotype in the Japanese and American Black populations [13, 14]. Moreover, in the Sugimoto et al [12] study concerning genetic influence on the response to a hypertensive drug, out of the eighteen genes studied, only the A-638-G polymorphism of the *RGS2* gene showed an association with the HT phenotype. Homozygosity to this polymorphism conferred the best response to azelnidipine—a studied antihypertensive drug. Additionally, Kamide et al [30] have established the association of exactly this polymorphism with significant intima-media thickening of the carotid artery both in hypertensive population and general population in humans. In our study, homozygosity for the A-638-G *RGS2* polymorphism was present relatively frequently—in above 50 % of cases, which could potentially be a therapeutic indication in HT treatment and atherosclerosis development. However, the aim of our study was to identify a hypertensive and non-dipping genotype, and such genotype has not been identified.

Our study does have some limitations, resulting from its design and availability of data. Firstly, we were not able to examine the patients for the presence of autonomic neuropathy. This complication is directly linked to non-dipping, and it is likely that at least some non-dipping patients had developed neuropathy before the ABPM examination. However, the database of patients enrolled in the study included general information about the possible manifestations of autonomic neuropathy, and none of the patients reported such symptoms. Secondly, we analyzed two major factors responsible for the development of neuropathy: duration of diabetes and metabolic control (expressed as HbA1c level) as continuous variables. It is possible that a threshold of, for example 10 years of 9.0 % of HbA1c would prove a strong risk factor and evidence an effect unseen in a model testing these variables as continuous ones. To account for that, we have performed ancillary analyses with models for HT and non-dipping risks testing HbA1c and duration of diabetes as dichotomous factors. However, since no evidence of any nonlinear effects for both variables were noted, we considered them as continuous in the final analyses. Thirdly, the study was underpowered to perform subgroup analyses in children differing by pubertal stage. Although this factor did not prove significant in univariate and multivariate analyses, it may be possible that in pubertal patients, different factors may determine the risk of HT or non-dipping than in other groups, due to rapid hormonal changes. Finally, the multivariate model for HT showed that it was more frequent in non-dippers. This may be interpreted in both directions—with non-dipping predisposing to HT or HT provoking a measurement bias for non-dipping due to increased nighttime BP. Nevertheless, since in our cohort more than 40 % of patients with HT showed dipping, and 42 % of patients without HT were non-dippers, we retained this factor in the analysis as both were not mutually exclusive.

Concluding, in our study we have proven that in subjects with good/suboptimal metabolic control of diabetes, some clinical factors can be associated with the development of HT: male sex, atherogenic lipid profile and the presence of non-dipping. Furthermore, the non-dipping phenomenon is mainly dependent on the duration of diabetes, which decreases the odds of persistent dipping. Despite the identification of groups with confirmed clinical HT/dipping relationships, suggesting our study population was representative of those groups, we have not managed to confirm that the genetic variations in the *ACE* genotype—insertion/deletion (rs17997552), rs1800764, rs4459609—and *RGS2* rs2746071 genotype could be connected with the regulation of the BP and lead to the disturbances in both HT and/or non-dipping. It is possible that we have not managed to elucidate any relationship due to the young age of our subjects or the sample size being too small to demonstrate a weak effect of genetic predisposition.

## Electronic supplementary material

Below is the link to the electronic supplementary material.

Supplementary material 1 (PNG 18 kb)

## Abbreviations

T1DM: Type 1 diabetes mellitus
ACE: Angiotensin 1-converting enzyme
RGS: Regulator of G-protein signaling
ABPM: Ambulatory blood pressure measurements
BP: Blood pressure
HT: Hypertension
