# Supplementary figures and images for: Non-dipping and arterial hypertension depend on clinical factors rather than on genetic variability of ACE and RGS2 genes in patients with type 1 diabetes
Source: Acta Diabetol. 2014 Feb 23;51(4):633–40. doi: 10.1007/s00592-014-0568-0 (PMC4127442; doi:10.1007/s00592-014-0568-0)

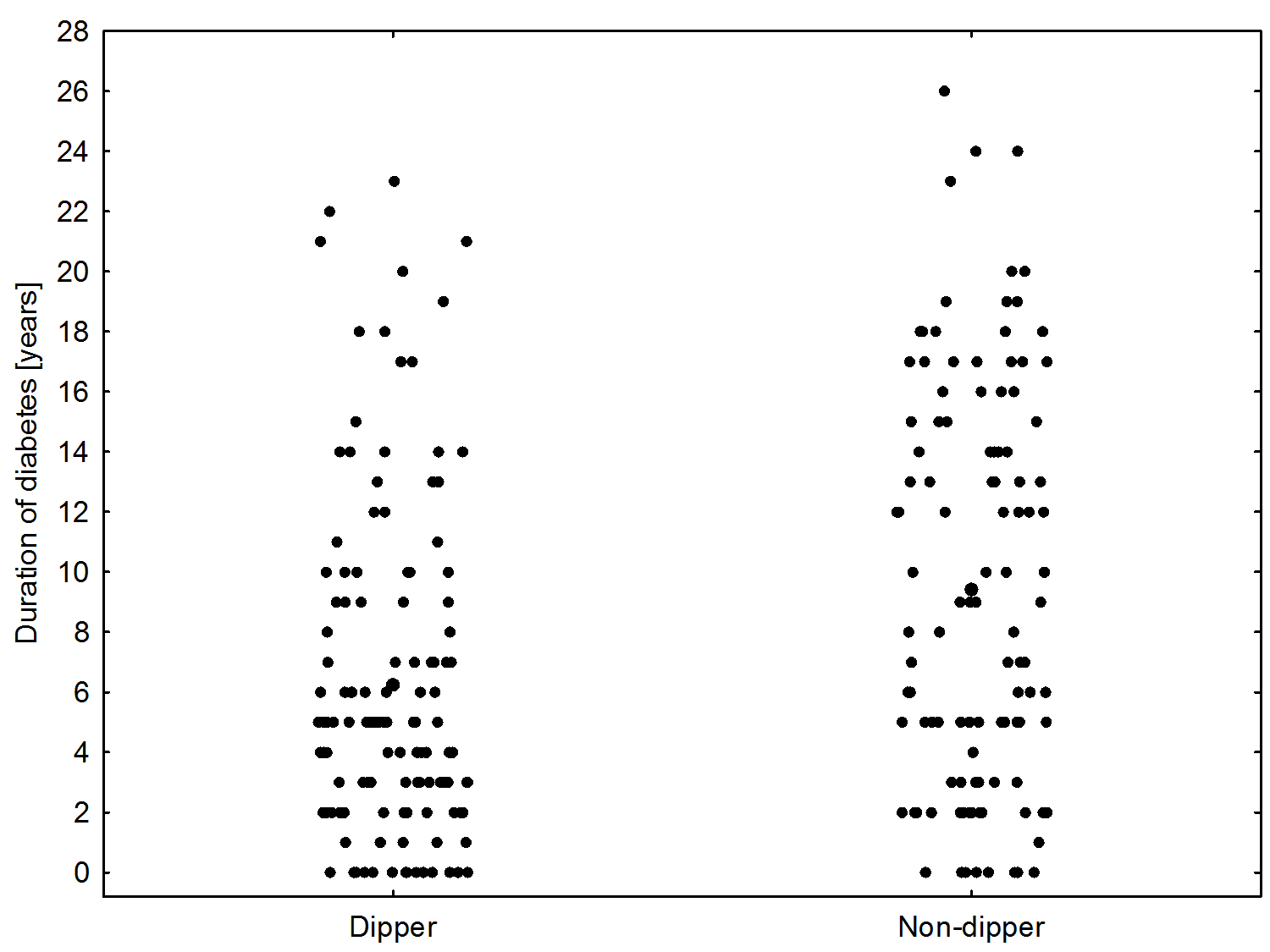

Supplement: Supplementary file 1 — Supplementary material 1 (PNG 18 kb) [file 592_2014_568_MOESM1_ESM.png]
